# Modern Drug Delivery Platforms Based on Photocrosslinkable Hydrogels (PCHs) in Dentistry: From Material Characteristics to Clinical Applications—A Review

**DOI:** 10.3390/ph19060837

**Published:** 2026-05-27

**Authors:** Susanna Sologova, Diana Sologova, Anna Shumkina, Vera Brazhnikova, Victoria Morozova, Sergey Sologov, Sergey Rusanov, George Anikin, Raisa Chilova, Elena Smolyarchuk, Elena Bakhrushina

**Affiliations:** 1 Department of Pharmacology, Sechenov First Moscow State Medical University (Sechenov University), Moscow 119991, Russia; sologova_s_s@staff.sechenov.ru (S.S.); smolyarchuk_e_a@staff.sechenov.ru (E.S.); 2 Department of Oral Surgery, Sechenov First Moscow State Medical University (Sechenov University), Moscow 119991, Russia; morozova_v_v@staff.sechenov.ru; 3 Sklifosovsky Institute of Clinical Medicine, Sechenov First Moscow State Medical University (Sechenov University), Moscow 119991, Russia; shumkina_a_a@student.sechenov.ru (A.S.); sergey.sologov@yandex.ru (S.S.); rusanov_s_n@staff.sechenov.ru (S.R.); anikin_g_s@staff.sechenov.ru (G.A.); chilova_r_a@staff.sechenov.ru (R.C.); 4 A.P. Nelyubin Institute of Pharmacy, Sechenov First Moscow State Medical University (Sechenov University), Moscow 119991, Russia; brazhnikova_v_i@student.sechenov.ru; 5 Department of Pharmaceutical Technology, Sechenov First Moscow State Medical University (Sechenov University), Moscow 119991, Russia; bakhrushina_e_o@staff.sechenov.ru

**Keywords:** hydrogels, drug delivery systems (DDS), regenerative dentistry, gelatin methacryloyl (GelMA), polyethylene glycol diacrylate (PEGDA), photocrosslinking

## Abstract

**Background/Objectives**: Modern dentistry increasingly requires biomaterials that not only replace damaged tissues but also actively regulate healing processes, modulate inflammation, and provide controlled delivery of therapeutic agents under the complex physicochemical conditions of the oral cavity. This review aims to analyze the potential of PCHs, particularly methacryloyl gelatin (GelMA), as multifunctional platforms for drug delivery in dental applications. **Methods**: This review provides a structured narrative synthesis of the literature, focusing on the physicochemical, biological, and translational aspects of photocrosslinkable hydrogels in dentistry. Special attention was given to the key functional requirements for hydrogels used in dentistry, including adhesion in a wet environment, antimicrobial properties, and the ability to provide sustained and localized release of active compounds. Natural, synthetic, and semi-synthetic polymers were comparatively evaluated to justify the selection of GelMA as a leading platform due to its tunable mechanical properties, biocompatibility, and photopolymerization capacity. The review also analyzes mechanisms of drug release activation and provides a comparative assessment of commonly used photoinitiators, including Irgacure 2959, lithium phenyl-2,4,6-trimethylbenzoylphosphinate (LAP), and camphorquinone, with emphasis on their cytocompatibility with oral tissues. **Results**: Applications of these hydrogels in endodontics, periodontology, peri-implantitis therapy, and regeneration of bone and dental pulp are summarized. **Conclusions**: Overall, photocrosslinkable GelMA-based hydrogels (PC-GelMA) represent promising multifunctional platforms for localized drug delivery and regenerative strategies in modern dentistry.

## 1. Introduction and Relevance

### 1.1. Literature Search Strategy

This review does not aim to meet formal systematic review criteria (e.g., PRISMA), but rather to provide a structured and critical synthesis of the literature. The literature search was conducted in the following electronic databases: PubMed (including MEDLINE), Scopus, Web of Science Core Collection, Google Scholar, ScienceDirect, and MDPI Open Access Journals (specialized search in Gels, Polymers, Materials, Pharmaceutics).

Two complementary search strategies were applied. The first strategy focused on photocrosslinkable or light-curable hydrogels based on GelMA and general PCHs using the query: (“hydrogel” OR “hydrogels” OR “GelMA” OR “gelatin methacryloyl”) AND (“drug delivery” OR “controlled release”) AND (“dentistry” OR “endodontics” OR “periodontitis”). The second strategy targeted synthetic PEGDA-based hydrogels for comparative analysis using the query: (“PEGDA” OR “polyethylene glycol diacrylate”) AND (“hydrogel” OR “scaffold”) AND (“dental” OR “oral”).

The search covered the period from 2000 to 2025. Considering the rapid progress in the field of PCHs, particular attention was paid to publications from the last five years (2020–2025); however, fundamental works from earlier periods were also included if they made a significant contribution.

Inclusion criteria: original research articles (in vitro, in vivo, ex vivo), systematic reviews, meta-analyses, clinical studies, English language, works dedicated to the development and application of photocrosslinkable hydrogels (especially GelMA and its composites) or PEGDA-based systems in the context of drug delivery in dentistry and the maxillofacial area.

Exclusion criteria: conference abstracts, editorial notes, comments, letters to the editor, articles without full text, publications with obvious signs of data duplication or low methodological quality (lack of control groups, insufficient statistics).

The review is limited by English-language publications, possible publication bias, and limited access to some articles from closed sources. However, the selected databases and search strategy confirm the representativeness of the current state of research.

### 1.2. Hydrogels in Medicine (Dentistry): Key Properties

Hydrogels are complex polymer systems characterized by matrices consisting of hydrophilic macromolecular structures. These macromolecules are interconnected by chemical, physical, or supramolecular interactions of amphiphilic units. Gelation of hydrogels can be caused by various external stimuli, including changes in temperature, pH, exposure to electric or magnetic fields, enzymatic activity, light exposure, and other external factors [1]. This class of materials possesses significant scientific potential in the context of biomedical applications, which makes it highly relevant for further research. Thus, hydrogels are widely employed in tissue engineering and regenerative medicine [2,3,4], female reproductive medicine [5], urology [6], rheumatoid arthritis therapy [7], and the treatment of intervertebral disc degeneration [8]. With the advancement of hydrogel technologies, increasing attention is being directed toward hydrogel microspheres, which are finding widespread application in medicine. They facilitate drug delivery, tissue engineering and regeneration, wound healing, and antitumor therapy [9]. Hydrogels have also found broad utility in dentistry and maxillofacial surgery. These materials are actively used for endodontic treatment [10], oral cancer therapy [11], and the regenerative engineering of the dentin–pulp complex and periodontal tissues [12].

Fundamental reviews on biomedical hydrogels exist [10,11,12,13,14], yet the translation of material innovations into dental clinical practice remains fragmented. Previous studies have often prioritized either narrow polymer chemistry [15] or broad regenerative medicine principles [4], neglecting the distinct oral microenvironment—microflora, biomechanics, and anatomy. The present review adopts an interdisciplinary approach, framing PCHs as adaptive platforms. Its novelty is defined by the systematic analysis of three parameters previously unexplored in a dental context:

1. The type of polymer base (natural, synthetic, hybrid) and its impact on bioactivity.
2. The mechanism of release activation (diffusion, degradation, external stimuli) and its correlation with specific dental pathologies.
3. Clinical adaptation (the ability of the material to function under conditions of constant moisture, chewing loads, and microbial colonization).

In contrast to previously published reviews, which focused on a single type of pathology [10,12] or a single class of compounds, this work offers a matrix for selecting a hydrogel depending on the target tissue (pulp, periodontium, bone) [11].

This review makes the following significant contributions to the literature:

Comparison of Release Mechanisms (Table 1): For the first time in dental applications, the advantages and disadvantages of pH-, enzyme-, and light-sensitive systems are systematically compared, focusing on their efficacy in oral conditions (pH variability, presence of saliva, limited light penetration depth).

Analysis of Photoinitiators (Section 4.2): The rationale for switching from UV-activated Irgacure to visible light-activated initiators (LAP, camphorquinone) is presented in terms of their cytocompatibility with human dental pulp cells (hDPSCs) and the depth of light penetration through dental hard tissue. The relationship between photoinitiator activation wavelength, polymerization depth in dental tissues, and biosafety within photocrosslinkable hydrogel systems has not yet been systematically discussed in the context of dental drug delivery applications.

The concept of a “multi-target therapeutic arsenal”: A classification of hydrogels based on their functional load (antibacterial, anti-inflammatory, proangiogenic) is proposed to advance the use of monotherapy in the treatment of chronic inflammatory diseases (periodontitis, peri-implantitis).

Thus, this review represents the structured study that not only lists the properties of GelMA but also establishes direct links between its chemical structure, physicochemical properties (porosity, swelling kinetics), and therapeutic effect (inflammation suppression, dentin regeneration). This allows us to propose a roadmap for the development of personalized delivery systems in dentistry, which is an important step in improving the quality of dental treatment.

### 1.3. Rationale for DDS Within Hydrogels

**Figure 1:**
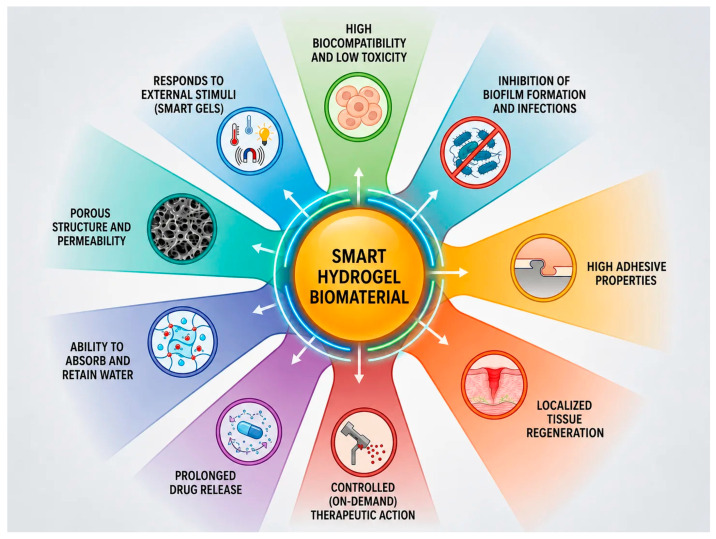
Key properties of hydrogels for their application as delivery systems: from biocompatibility and adhesion to controlled release and stimuli-responsive (“smart”) behavior.

The transition from passive materials to active, intelligent therapeutic platforms is no longer merely desirable—it critically depends on the development of hydrogel-based delivery systems. In dentistry, the growing adoption of these materials reflects their unique and precisely tunable properties, which can be tailored to meet the distinct demands of the oral environment. The information that is prescribed in Figure 1 provides a schematic representation of a “sun” diagram illustrating the multifunctional requirements of hydrogel-based drug delivery systems (HDDS) in dentistry. The central core represents the multifunctional smart hydrogel platform. The surrounding rays are grouped into key property sectors: biocompatibility and low toxicity, biofilm inhibition/Anti-biofilm activity, high adhesion, localized tissue regeneration, controlled (on-demand) action, prolonged drug release, high water-absorption capacity (swelling), porosity and permeability, stimuli responsiveness (“smart” gels).

### 1.4. Existing Approaches to the Activation of Pharmacological Agent Release and Photoactivation

The rational design and synthesis of hydrogels are fundamental for creating responsive hydrogel-based drug delivery platforms. Apart from the biocompatibility, biodegradability, mechanical and structural stability, one of the most crucial things for a drug delivery system is stimuli-responsiveness [16]. The ability to adopt characteristics in relation to physicochemical stimuli enhances efficacy, optimizes bioavailability, and reduces adverse effects of the drug [17]. Consequently, an on-demand drug delivery system is developed, also referred to as intelligent biomaterial.

External-stimuli-responsive-based hydrogels show significant potential for drug delivery in dental tissues [18,19]. Despite the remarkable therapeutic outcomes achieved with externally stimulus-responsive drug delivery platforms, several limitations remain to be addressed. The subsequent sections examine these issues, organized by the type of stimulus.

Hydrogels can be engineered to exhibit sensitivity to stimuli, including pH, light, redox conditions, temperature, glucose, and enzymatic activity; the advantages and disadvantages of each activation type are summarized in Table 1.

**Table 1:** Comparison of Stimuli-Responsive Mechanisms for Drug Release in Dental Applications.

Activation Type	Advantages	Disadvantages	Quantitative Performance Metrics
pH-sensitive	Ability to react in site-specific conditions; post-gelation adhesive performance; reduced bacterial biofilm burden [20,21].	Poor stability in the high-humidity oral environment (premature softening and degradation) [20]; variability in performance due to highly pH-sensitive degradation rate [20]; high variability of pH levels in the periodontal environment [22].	pH range: 5.5–7.5; swelling ratio ≈600% (1% ZIF-8) and >500% (3–5% ZIF-8); ~90% swelling reached within 6 h; drug release: 70% (pH 5.0), 58% (pH 6.5), 47% (pH 7.5) (60 h) (for Targeted Therapy of Periodontitis) [23].
Photosensitive	High sensitivity, fast response speed, high spatial resolution [24].	Limited efficacy in deep tissue regions or areas that are difficult for light to reach [25].	Irradiation wavelength: 440–490 nm (LED)/387 nm (photoinitiator); gelation time: 20–40 s; curing time: up to 120 s; effective curing depth: 2–5 mm; cell viability >80% [26].
Redox-sensitive	Antibacterial and antioxidant/anti-inflammatory effects in one platform [26].	Limited activation outside ROS-rich sites [27]; ROS-triggered bond cleavage can compromise structural integrity [26].	Oxidative degradation: borate ester bond cleavage leads to a reduction in H_2_O_2_ scavenging, reaching <65% within 1 h and <100% within 12 h [28].
Enzyme-sensitive	Periodontitis is associated with increased protease, lipase, and glycosidase activities in saliva; bacterial enzymes (e.g., Porphyromonas gingivalis gingipains) serve as specific triggers [29].	Dependency on collagenase presence for degradation was noted as technical limitations [30]; enzyme-sensitive groups are prone to non-specific hydrolysis under high-temperature and high-humidity conditions [31]; freeze–thaw cycles during storage may induce conformational changes in enzyme-responsive moieties, compromising their functionality [32].	Surface properties: high specific surface area (~1163.9 m^2^/g) with high adsorption capacity. Degradation (lysozyme-mediated): ~21.2% (10 μg/mL) vs. ~3.5% (2 μg/mL) for CS; reduced to ~14.7% and ~1.5% for modified CS-Str hydrogels [22,33].
Temperature-sensitive	Biphasic release (initial burst followed by sustained release up to 15 days) [22]; accurate and prolonged local delivery after gelation [23].	PF127-based hydrogels are prone to instability in the aqueous phase [34]; slow gelation (up to ~20 min) [35]; presence of ions in the oral microenvironment can alter the thermoresponsive behavior [36]; local temperature within the periodontal microenvironment varies according to anatomical location [37].	Sol–gel behavior: thermoresponsive (30–37 °C); liquid at low temperatures (4–25 °C); rapid gelation at 37 °C (~<1–5 min). Stability/degradation: prolonged stability (≥6 months at 4 °C); delayed degradation/dissolution (>9 days–8 weeks, initial faster phase followed by slower). Drug release: biphasic—initial burst (~50% in 3 days) followed by sustained release (up to ~80% by 21 days; some systems ~72 h sustained release) [38,39,40]. Viscosity: low (<500 cP), ensuring injectability [40].
Glucose sensitive materials	Synchronously intervenes in ROS burst, chronic inflammation, and pathogenic biofilm formation [41].	Variability in gel stability and manufacturing reproducibility [42].	Gelation: Gel time decreases from >1900 s to <200 s with increasing crosslinking. Swelling: increases with glucose concentration (GC). Drug release: glucose-dependent <15% (0 mg/dL) vs. >85% (250 mg/dL). Dynamic response: adaptive release (e.g., ~65% at 140 mg/dL vs. ~78% at 200 mg/dL). Antibacterial activity: enhanced at higher GC (greater drug release) [43].

**pH-sensitive hydrogels**

pH-sensitive degradable hydrogels represent a class of smart biomaterials that can cleave covalent bonds in response to variations in environmental pH, leading to their degradation [44]. These systems are designed to respond to pathological pH shifts, thereby facilitating site-specific therapeutic delivery.

There are two principal design strategies.

The first involves employing polyelectrolytes that can undergo protonation or deprotonation in response to pH changes, such as materials with amine or carboxyl functional groups. Deprotonation-responsive systems enable modulation of drug release in alkaline periodontal microenvironments [45].

The second strategy relies on the incorporation of acid-labile linkages within the polymer network. These bonds cleave under acidic pH, enabling targeted degradation and drug release. A potential limitation of acid-labile linkages is their limited chemical stability, which may lead to off-target or premature therapeutic release [46].

**Temperature-sensitive hydrogels**

Thermosensitive hydrogels are an advanced class of injectable biomaterials capable of undergoing sol–gel phase transition at physiological temperature [15].

The application of thermosensitive hydrogels presents a novel and promising avenue for periodontal regeneration, offering conforming to defects, sustaining the release of therapeutic agents, and creating bioactive microenvironments conducive to tissue repair [47]. Bakuchiol-loaded thermosensitive hydrogel (BTH) has been developed as a local drug delivery system for periodontitis management [48].

Phase transformation from sol to gel occurs because of temperature-induced changes in the interactions between polymer hydrophilic/hydrophobic domains and water. Hydrogels can form either above the lower critical solution temperature (LCST) or below the upper critical solution temperature (UCST), depending on the specific composition and ratio of hydrophilic and hydrophobic components [49]. While natural thermoresponsive polymers exhibit weak mechanical strength and slow temperature responses, synthesized polymers offer greater adjustability in physical properties [50].

**Redox-sensitive**

The redox-responsive behavior of the hydrogel is achieved through the incorporation of specific chemical moieties [51]. Redox-responsive materials exhibit microenvironment-specific responses via bond cleavage or structural changes under oxidative/reductive conditions [45]. These materials stimulate osteogenesis and bone formation, mitigate inflammatory responses, and improve regeneration of periodontal soft and hard tissues [52]. ROS-responsive HP-PVA hydrogel based on boronate ester bonds has been reported to undergo bond cleavage in the oxidative microenvironment of periodontitis, enabling controlled release of antibacterial and anti-inflammatory agents and reducing alveolar bone resorption [53].

**Glucose-sensitive materials**

Glucose-responsive materials are smart systems capable of dynamically sensing fluctuations in glucose concentration. Their core mechanism relies on enzymatic catalysis or glucose-mediated structural transformations [45].

This strategy is significant in diabetic periodontitis, characterized by hyperglycemia, excessive ROS accumulation, and amplified inflammatory responses [54]. Glucose-responsive hydrogels utilize the hyperglycemic microenvironment characteristic of diabetic periodontitis as a physiological signal that activates controlled drug delivery [55].

**Enzyme-sensitive materials**

Enzyme-responsive materials represent a class of intelligent biomaterials that rely on “enzyme-substrate” molecular recognition mechanisms to induce structural transformation or functional activation. Drug release is commonly controlled via enzyme-cleavable motifs and the construction of enzyme-activated prodrug systems. Under the action of target enzymes, enzymatic activity induces bond cleavage or structural changes to trigger drug release [56].

In periodontitis, elevated levels of matrix metalloproteinases and other degradative enzymes provide accessible targets for the development of enzyme-responsive delivery systems [57]. An MMP-8-sensitive polyethylene glycol hydrogel capable of releasing antimicrobial agents, including minocycline, has been proposed as a localized delivery system for periodontal therapy [58].

**Endogenous electricity-sensitive materials**

Endogenous bioelectric signals in periodontal tissues modulate cellular behavior through alterations in membrane potential and ion fluxes, including Ca^2+^, Na^+^, K^+^, and Cl^−^ [45]. Activation of these downstream signaling cascades ultimately promotes the proliferation and differentiation of fibroblasts, osteoblasts, and periodontal ligament cells [46]. Electrically responsive biomaterials can guide the directed migration of cells involved in periodontal regeneration to the injury site, accelerating the healing process [59].

Conductive hydrogels incorporating poly (3,4-ethylenedioxythiophene) (PEDOT) have been reported to facilitate the transmission of endogenous bioelectrical signals, promoting cellular alignment and increased calcium ion influx, which may contribute to periodontal tissue regeneration [53]. A piezoelectric hydrogel composed of BaTiO_3_ nanoparticles embedded in a gelatin matrix has been developed to generate bioelectrical stimulation and enhance periodontal tissue regeneration in periodontitis models [60].

**Multi-sensitive materials**

Multi-responsive hydrogels integrate two or more stimuli-responsive mechanisms to achieve enhanced precision and therapeutic efficiency.

A dual-responsive hydrogel formulation containing “intelligent particles” designed to eliminate cariogenic biofilms was developed. The hydrogel forms within one minute at physiological temperature, exhibits strong mucoadhesive properties for prolonged oral retention, and rapidly disintegrates under acidic conditions—triggering acid-responsive antibacterial and biofilm-disruption activity [61].

**Photosensitive materials**

Light-responsive materials include photothermal agents, photosensitizers, and photocatalysts. Photothermal materials absorb light to trigger localized surface plasmon resonance (LSPR) or molecular vibration relaxation. They efficiently convert light energy into heat [55]. Photosensitizers, under specific wavelength light irradiation, can produce ROS. This occurs through electron transfer interactions with surrounding oxygen molecules [19]. Further details regarding photocatalytic materials are provided in Section 4.

The next chapter applies these activation strategies to the selection of suitable hydrogel materials, focusing on GelMA as a clinically adaptable platform.

## 2. Selection Criteria and Material Platforms for Photocrosslinkable Hydrogels

When restoring bone tissue damaged by periodontal disease, traditional materials such as autogenous bone, allografts, and xenogenic bone are commonly used [62,63]. Autogenous bone offers complete histocompatibility and high bioactivity, which ensures optimal bone regeneration [64]. However, bone harvesting results in a secondary wound at the donor site and increases the risk of postoperative complications, such as infection, wound dehiscence, and increased bleeding. Allografts provide high availability in terms of variety [65], as well as osteoinductivity and osteoconductivity [66]. However, allogenic bone is associated with the risk of immune rejection and requires careful donor selection and immunosuppressive therapy. Despite its widespread use in dental practice, availability, and osteoinductivity, xenogenic bone has several drawbacks. Due to species differences, xenografts are less biologically active and capable of providing mechanical support.

PCHs, through the mineralization of amorphous calcium phosphate, restore defects without the need for transplants. It features a hypoallergenic composition and high biocompatibility. The hydrogel also exhibited superior mechanical properties, including enhanced underwater tissue adhesive strength and compressive resistance [67]. After adding HAP, the hydrogel composites show significant improvement in mechanical properties, structural and compositional similarities to natural bone ECM, and promoted biocompatibility by preserving cellular viability and enhancing cellular proliferation [68].

Several inherent properties of resin composite render it a more advantageous restorative material relative to many alternatives. In addition to having high adhesion and a wide color range, they can be virtually invisible, which is especially important when treating cavities on anterior teeth. Depending on whether they are filled with macrofill or microfill particles, composites can serve different functions. Microfill composites are easier to polish, which is important from an aesthetic standpoint. On the other hand, macrofill composites are more durable and are more commonly used for restoring molars [69,70]. Typically, dental composites used in restorative procedures exhibit volumetric shrinkage ranging from less than 1% up to 6%, depending on the formulation and curing conditions. Polymerization shrinkage stress of resin-based materials has been related to several unwanted clinical consequences, such as enamel crack propagation, cusp deflection, marginal and internal gaps, and decreased bond strength [71]. Also, when filling teeth, the polymerization reaction only completes about 70%. As a result, triethylene glycol dimethacrylate (TEGDMA) and 2-hydroxyethyl methacrylate (HEMA) accumulate and seep through the dentin into the pulp. These two monomers are commonly found in dental adhesives and filling materials. Through the pulp, TEGDMA or HEMA enters the bloodstream, affecting numerous cells. TEGDMA was found to inhibit the formation of Pakeratinocyte layers and diminish their viability and adherence, thus negatively affecting oral wound healing [72].

In addition to GelMA’s reduced cytotoxicity and high adhesion, it exhibits negligible shrinkage (<0.5%) during photocrosslinking. The hydrophilic gelatin backbone maintains hydration equilibrium, preventing the dense polymer chain collapse seen in TEGDMA systems [73,74].

Over the past three decades, dentists have preferred calcium silicate-based materials for direct pulp capping of tooth stumps, with particular clinical attention given to mineral trioxide aggregate (MTA) and biodentine (BD). Thanks to the properties of tricalcium silicate cement MTA, its high pH level, the release of calcium ions, and its ability to form hydroxyapatite at the dentin interface contribute to the predictable formation of a dentin bridge, making it the benchmark material against which new products are compared. The disadvantages of MTA, such as long setting times, color change potential, and difficulty of use, have been addressed in BD through additives that reduce setting time and improve consistency. However, both of these bioactive cements induce initial inflammation due to their high alkalinity (pH > 10) and have low mechanical strength [75].

The neutral pH (7.2) of the GelMA/LAP formulation prevents the initial inflammatory response observed with alkaline pastes. No significant increase in proinflammatory cytokine levels was detected in hDPSCs, confirming the excellent biocompatibility of the PCHs for pulp regeneration. In addition, thanks to the covalent 3D network formed by methacryloyl groups during photopolymerization, the hydrogel has high mechanical stability [74].

### 2.1. Hydrogel Materials

The base of hydrogels can consist of natural, synthetic, or semi-synthetic polymers. Depending on the chosen material, the physicochemical properties, biological activity, and consequently the area of application may vary. In Table 2, examples of various photosensitive polymers and their properties are briefly discussed.

The main advantage of natural hydrogels is their high biocompatibility, which translates into properties such as high hydrophilicity, biocompatibility, and strong cellular adhesion. However, natural polymers do not polymerize under light due to the lack of photoreactive groups [76]. For this reason, they are not included in Table 2. In turn, synthetic hydrogels can be photopolymerized due to the presence of acrylate groups with double bonds, enabling photopolymerization through a free radical chain reaction [77]. Yet, aside from other physicochemical properties listed in Table 2, which are certainly advantages, such as high mechanical strength and minimal immunogenicity. Synthetic hydrogels have a drawback that affects their application potential: low biological activity. Therefore, we need a material that can photopolymerize while incorporating the positive properties of both groups. Hybrid hydrogels possess such characteristics.

**Table 2:** Comparative characteristics of different types of hydrogels.

Type of Hydrogel	Synthetic	Semi-Synthetic
Material	PEGDA [78], MHA [30].	GelMA [79], GelMA/PEGDA [80], PEG-Fibrinogen [81].
Degree of modification	DoA (Degree of Acrylation) >90% [82].	DoM (Degree of Methacryloylation) 57–80% [83].
Key physicochemical properties	High mechanical strength [84], Minimal immunogenicity [85].	High adhesion, mechanical strength, biocompatibility, hydrophilicity [85].
Biological activity	Low [84].	High [63].
Applications in dentistry	GBR barriers (Guided Bone Regeneration) [74].	Pulpitis [86]; Periodontitis [34]; Peri-implantitis [35].

### 2.2. Advantages and Limitations of Different Hydrogel Types

We examined the nuances in the operation and use of different types of hydrogel bases. Among PCHs, GelMA, a semi-synthetic polymer, is the most widely used for several reasons discussed in Table 3.

### 2.3. GelMA as a Leading Hydrogel Platform

In Table 3, we can see that GelMA, being a hybrid hydrogel, combines the qualities of both natural and synthetic hydrogels. Natural hydrogels lack the ability to polymerize, while synthetic ones can have a cytotoxic effect due to the photo-initiators used, such as Irgacure [87]. GelMA, with its methacryloyl groups, has the property of photo-crosslinking. In this process, the photo-initiator LAP is used, which maintains cell viability. Additionally, LAP (Lithium phenyl-2, 4, 6-trimethylbenzoylphosphinate) is water-soluble, fully dissolving in GelMA to ensure uniform polymerization without agglomerates [74]. The extensive hydration of natural hydrogels promotes bioactivity and cell adhesion, but compromises mechanical strength. The methacryloyl groups provide a covalent network, while the RGD sites from collagen offer high adhesion and bioactivity. Moreover, the presence of collagen MMP in GelMA ensures degradation that is dependent on the environment and prolonged release of the drug [73]. With its low viscosity and rapid in situ curing, the balance of natural and synthetic materials gives GelMA good injectability and minimal invasiveness. These advantages allow us to use GelMA in pulpitis [34], periodontitis [34], and peri-implantitis [35].

One of the limitations of GelMA is that its mechanical properties are primarily adjustable through the degree of methacrylation (DOM/DS), which may restrict the fine-tuning of other interdependent parameters such as porosity, viscosity, and elasticity. A high degree of substitution (DS > 70%) provides stiffness, but a dense network reduces porosity and, consequently, swelling. Conversely, a low DS (< 50%) is too soft and fails to retain shape. Additionally, gelatin obtained through the hydrolysis of collagen from various sources exhibits different physical and chemical properties. Due to all these drawbacks, GelMA has high variability from batch to batch, which limits its applications, for instance, in orthopedic dentistry (crowns, bridges, chewing fillings), where predictability and long-term stability are crucial [88].

PEGDA has a broader range of mechanical properties (tunable from 5 kPa to over 70 kPa) and high batch reproducibility due to the absence of a natural base. PEGDA can be used in thin GBR barriers (implantation, 0.1–0.5 mm) due to its high stiffness and lack of degradation in saliva [17]. In contrast to GelMA, which experiences softening (<30 kPa to <10 kPa over 2 weeks) [89], PEGDA maintains mechanical stability up to 1.7 MPa in a composite, making it suitable for bone defects [90].

Additionally, PEGDA is bioinert due to the lack of cell-binding ligands. This allows for its use in antibacterial coatings for implants, unlike GelMA, which has a natural base that can lead to biofilm formation [17].

Thus, the specific properties of PEGDA and GelMA play different roles depending on the desired application.

We consider GelMA as the main example and leader because there are more preclinical studies on it than on synthetic or other semi-synthetic PCHs [91].

## 3. Comparative Analysis of GelMA, PEGDA, and Hybrid Systems

A comparative evaluation of GelMA, PEGDA, and hybrid hydrogel systems could be conducted based on key biomaterial parameters, such as mechanical properties, adhesive behavior, biocompatibility and biosafety, biodegradation and its controllability, and sterilization and storage stability. These parameters determine materials’ suitability for different tissue engineering applications.

### 3.1. PEGDA

Synthetic hydrogels, e.g., poly (ethylene glycol) (PEG), poly(vinyl alcohol) (PVA), and poly(2-hydroxyethyl methacrylate) (PHEMA), boast controllable degradation and microstructure, and are generally suitable in terms of mechanical strength but lack biological components [92].

PEGDA possesses photocrosslinking properties, low viscosity, and high solubility, and is obtained through chemical modification of polyethylene glycol (PEG), which exhibits high biocompatibility and minimal immunogenicity [93].

PEG hydrogels typically exhibit minimal or no intrinsic biological activity due to the nonadhesive nature of PEG chains [78].

Despite its many advantages, PEGDA is generally inelastic and brittle, which makes it more likely to be used in combination with other materials rather than alone for bone tissue engineering [94].

PEGDA hydrogels showed minimum cell adhesion and spreading. PEGDA hydrogels with incorporation of RGD-PEGMA exhibited significantly higher cell adhesion and spreading [78].

### 3.2. GelMA

Naturally derived hydrogels, e.g., gelatin, collagen, chitosan, and hyaluronic acid (HA), are widely used in biological applications because of their cell signaling abilities, cellular interactions, and biodegradability. However, these materials are not always ideal for tissue engineering applications and can be limited by, for example, low mechanical strength, uncontrolled structural degradation, or potential immunogenicity [92].

Such cases require chemical modifications to the natural hydrogel.

First, gelatin, a natural hydrogel, is a hydrolysate of collagen [95] but with significantly lower immunogenicity [12] due to a lower number of aromatic groups [32]. Second, gelatin contains a variety of bioactive motifs, including arginine–glycine–aspartic acid (RGD), which promotes the adhesion and growth of different cell types [37,96], and matrix metalloproteinase (MMP), which is used for cell remodeling and further enhances the physicochemical properties of gelatin hydrogels [97].

GelMA hydrogels typically contain less than 5% methacrylic anhydride (MA) [21]. Most of the functional amino acid motifs of gelatin, including RGD and MMP, are not significantly affected, thereby retaining the cell adhesion properties of gelatin in GelMA materials.

GelMA provides significant flexibility in adjusting its properties through modifications in its synthesis and processing, such as variations in crosslinking conditions.

It is known that higher substitution rates in GelMA could lead to a higher density of crosslinking sites that influence the viscoelasticity of the resulting GelMA network. The extent of substitution could affect properties such as porosity, mechanical stiffness, and swelling behavior of the hydrogel.

The stiffness of GelMA hydrogel can be adjusted by controlling the crosslinking degree, but this is limited because the active groups on the gelatin chains, which can react with MA, represent less than 5% of the total amino acids. Additionally, as the degree of crosslinking increases, steric hindrance becomes more significant, which interferes with the crosslinking reaction [98].

The excellent biocompatibility of GelMA hydrogels makes them suitable as cell culture matrices that mimic native ECM [85]. Encapsulated cells are capable of adhesion, spreading, migration, and proliferation within the hydrogel matrix [99,100]. GelMA retains the intrinsic bioactivity of gelatin due to the preservation of cell-adhesive arginine–glycine–aspartic acid (RGD) motifs and matrix metalloproteinase (MMP)-sensitive sequences [101]. The methacryloyl modification usually affects less than 5% of the amino acid residues, ensuring that most of the functional domains remain intact [102]. Additionally, GelMA hydrogels are still vulnerable to enzymatic degradation by collagenases (MMP-1 and MMP-8), which further supports the retention of cell-remodelable motifs [103,104]. High cell viability (>80%) is generally observed in photocrosslinked cell-laden GelMA hydrogels [76].

### 3.3. Hybrid Systems

Hybrid GelMA–PEGDA systems are designed to integrate mechanical reinforcement from PEGDA with biological functionality from GelMA to provide a favorable environment for tissue regeneration. These systems are particularly advantageous in wound healing, drug delivery, and tissue engineering [103,104]. Pdlsc-loaded GelMA/PEGDA hydrogels with different compositions resulted in the formation of robust new bone in the defects compared with the control group [103]. A new GelMA/PEGDA/F127DA bioink not only possesses satisfactory mechanical properties, but also an appropriate degradation rate that is more compatible with the time course of bone regeneration, guiding the new bone tissue to grow inside the defect when implanted in vivo. In conclusion, hybrid hydrogels provide a new approach to the clinical treatment of various dental conditions. Although hybrid hydrogels are mentioned in dental applications, the research available is far more limited compared with the extensive data on single-component hydrogels. The comparisons of GelMA, PEGDA and hybrid systems are presented in the Table 4.

## 4. Activation Strategies for Pharmacological Agents Within GelMA and Photoinitiation

### 4.1. Principles of GelMA Photopolymerization

GelMA is a photocrosslinkable material, and its ability to undergo light-induced polymerization allows for precise control over the hydrogel’s structure and mechanical properties. One of the key methods used to create GelMA-based scaffolds is photopatterning, where photomasks are employed to impart a specific topography or to engineer 3D architectures. Light irradiation passes only through the transparent areas of the photomask, inducing crosslinking in the underlying GelMA solution and imprinting the desired patterns on the hydrogel structure [110].

GelMA’s photocrosslinking is facilitated by the presence of unsaturated photocrosslinkable groups, particularly primary amine (-NH2) and hydroxyl (-OH) groups. Primary amine (-NH2) and hydroxyl (-OH) groups are mainly involved in this substitution reaction, where methacryloyl groups are introduced onto gelatin [85]. The polymerization of GelMA occurs in an aqueous state by a free radical mechanism in the presence of a photoinitiator [111].

One of the key external factors influencing the mechanical properties of GelMA hydrogels is the concentration of the photoinitiator, which plays a critical role in controlling and adjusting the hydrogel’s properties [112].

Prolonged UV exposure can lead to the formation of free radicals, resulting in DNA damage and impairing cellular function, which is a significant limitation for its application in medical and dental fields.

To overcome this, visible light photoinitiators, such as lithium acylphosphinate salt (LAP), have been investigated. LAP demonstrates high water solubility and allows for effective polymerization at lower photoinitiator concentrations and longer light wavelengths (405 nm), as compared with the more commonly used Irgacure 2959 [113]. Importantly, visible light is expected to cause less cellular damage and penetrate tissues more efficiently, enabling a greater depth of cure. This shift to visible light photopolymerization offers potential advantages for hydrogels in dental applications, where the protection of surrounding tissues and cells is paramount [114].

Compared with other methods, photopolymerization displays numerous benefits, such as injectability, rapid gelation, improved mechanical properties, and suitability for customized bioprinting, along with easy incorporation with various cell types. However, the free radicals generated during crosslinking can attack cell membranes and result in cell death [76].

### 4.2. Major Photoinitiators

Among the most commonly used free radical photoinitiators are 2-hydroxy-4′-(2-hydroxyethoxy)-2-methylpropiophenone (IC-2959), lithium phenyl-2, 4, 6-trimethylbenzoyl phosphinate (LAP), and camphorquinone due to their excellent biocompatibility and minimal immunogenicity [115,116].

Irgacure 2959 (I2959) is the most widely used photoinitiator for cell encapsulation and tissue engineering applications due to its high free radical generation efficiency and relatively higher water solubility (below 2%) [117]. Irgacure 2959 is well tolerated by various cell types, including chondrocytes (BC), mesenchymal stem cells (gMSC, hMSC), corneal epithelial cells (SIRC), human embryonic germ cells (LVEC), and osteoblasts (hFOB) [118]. Cells that proliferated faster are more sensitive to photoinitiator-induced toxicity, while cells with slower division rates showed lower toxicity [118]. Despite its popularity, it has some limitations, such as low water solubility and the need for UV light exposure (365 nm) for activation [74]. Its maximum absorption is at 276 nm and, due to its poor absorption, Irgacure 2959 requires extended exposure time [117].

The visible light photoinitiator LAP (405 nm) is a possible alternative for UV photoinitiator Irgacure 2959 (365 nm). Lithium phenyl-2, 4, 6-trimethylbenzoyl phosphinate (LAP) has emerged as a preferable photoinitiator for many biological applications, including dentistry [74]. The increased water solubility and better polymerization kinetics of LAP enable cell encapsulation at lower concentrations and reduced light intensity, which reduces the associated toxicity and enhances cell viability [117]. Studies have shown that LAP does not affect cell viability when exposed to inactivated conditions, in contrast to Irgacure 2959, which causes significant cell toxicity under UV exposure [74]. Cell survival in gels polymerized with 1 min exposure to 10 mW/cm^2^ of 365 nm light exposure and 2.2 mM LAP was statistically similar to a 6 min polymerization with the same molar initiator concentration of I2959 (corresponding to 0.05 wt%) at the same light intensity. Likewise, a concentration of 0.22 mM LAP combined with 10 min of light exposure results in comparably high cell survival [113]. LAP-containing GelMA hydrogels polymerized with a dental curing light set after 5 s of exposure, whereas I2959-containing hydrogels exposed to 365 nm UV light required at least 10 s for polymerization and did not form stable gels after 5 s [74].

Camphorquinone (CQ) is a photoinitiator commonly incorporated in most contemporary dental composite materials, including both dental composites and dental adhesives (DAs) [119]. CQ uses the visible light-curing systems to initiate the polymerization process [120]. When irradiated with visible light in the range of 460–480 nm, CQ generates free radicals, one of the major forms of reactive oxygen species (ROS), in the presence of coinitiators such as tertiary aromatic amines. However, CQ has its drawbacks, including the potential for leaching into the oral cavity post-polymerization, with concentrations up to 14 mmol/L potentially being released [121].

Photoinitiation is one of the most studied and widely used methods for activating the release of pharmacological agents in dentistry. From tissue regeneration to disease treatment, photoinitiation allows for the effective delivery of active substances to targeted areas with minimal side effects. Overall, LAP appears to offer a favorable balance between polymerization efficiency and cytocompatibility for cell-laden dental hydrogels, whereas I2959 is limited by UV activation and lower curing efficiency, and CQ is advantageous for compatibility with clinical blue-light curing systems but may present cytotoxicity concerns related to coinitiator use and leaching. However, direct comparisons among these photoinitiators under identical clinical dental curing-lamp conditions remain limited.

**Figure 2:**
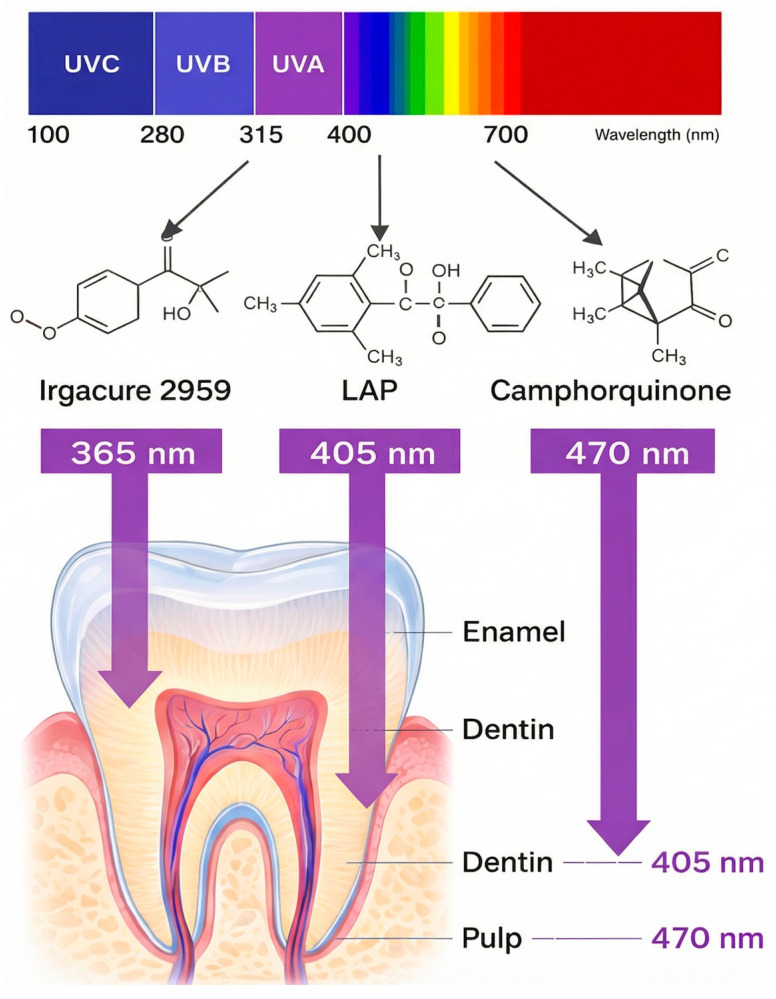
Schematic representation of the photoinitiators Irgacure 2959, LAP, and camphorquinone, their activation wavelength ranges, and the corresponding depth of light penetration in dental tissues. The penetration depth is shown qualitatively: 365 nm light is mainly associated with enamel-level penetration, 405 nm with deeper penetration into dentin, and 470 nm with the deepest penetration toward the pulp region. The scheme is not intended to represent exact quantitative penetration depths.

The clinically significant relationship between the wavelength of light used for photopolymerization, photoinitiator activation, and light penetration into dental tissues is illustrated in Figure 2.

## 5. Therapeutic Effects of Hydrogels

**Figure 3:**
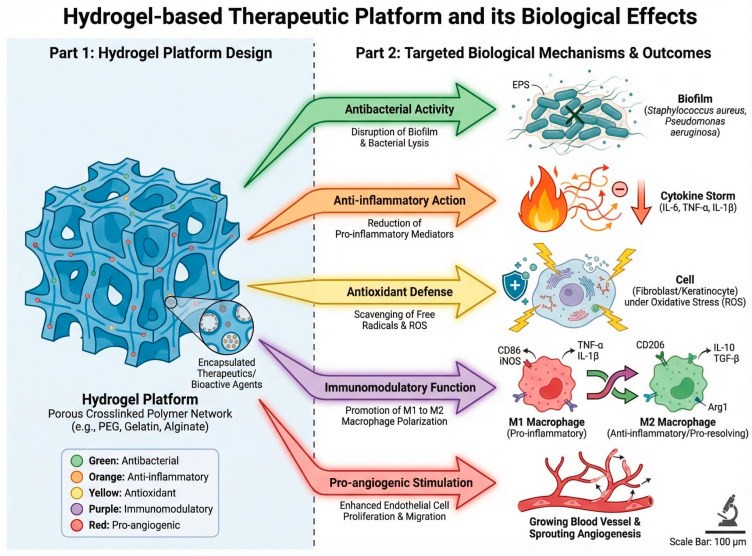
Schematic representation of a multifunctional hydrogel platform as a therapeutic arsenal against oral pathologies.

The hydrogel serves as a central delivery system, releasing various bioactive agents to simultaneously target five key pathological processes: bacterial biofilms (antibacterial action), inflammation (anti-inflammatory action), oxidative stress (antioxidant action), immune dysregulation (immunomodulatory action), and insufficient vascularization (pro-angiogenic action) as summarized in Figure 3. This multi-targeted approach is essential for comprehensive tissue regeneration in the treatment of complex oral diseases such as periodontitis, pulpitis, and peri-implantitis.

### 5.1. Description of Hydrogels with Anti-Inflammatory Effects

Contemporary research in regenerative medicine is centered on developing hydrogels with multiple functions. These hydrogels are designed to support tissue regeneration and simultaneously control inflammatory responses. The main component employed in these investigations is GelMA, known for its photocrosslinkable properties.

An injectable GelMA hydrogel containing DEX-loaded halloysite clay nanotubes (HNTs). Dexamethasone (DEX) is a versatile medication known for its anti-inflammatory and mineralocorticoid effects. The gradual release of DEX over a 7-day period revived alkaline phosphatase activity and mineralization. In vivo results demonstrate accelerated bone formation after 6 weeks, with minimal localized inflammation noted after 7 days [122]. A two-layer fibrous scaffold made up of a 10% (weight-to-volume) GelMA layer infused with Ibuprofen (IBP) and a 20% GelMA layer incorporating amorphous magnesium phosphate (AMP). IBP is employed for anti-inflammatory purposes. The IBP layer effectively suppressed the release of IL-1α, TNF-α, and IL-6, as well as blocking NF-κB activation, all without causing any cytotoxicity. This versatile scaffold manages inflammation and directs tissue regeneration simultaneously [123]. PC-GelMA nanofibers with Aloe vera (AV), which acts as a natural antimicrobial and anti-inflammatory agent. GelMA/AV (70:30) nanofibers exhibited sustained antibacterial activity over 14 days and anti-inflammatory properties that favor healing [124]. Baikalin, which is part of the hydrogel [125], has antioxidant and anti-inflammatory properties. It effectively reduces the level of intracellular reactive oxygen species and suppresses the production of pro-inflammatory cytokines, including TNF-α, IL-1α, and IL-6, depending on the dosage.

### 5.2. Description of Hydrogels with Antibacterial Activity

Antibacterial hydrogels are medical materials engineered to eradicate microbes while promoting tissue regeneration. GelMA is frequently employed as their foundation because of its ability to undergo photocrosslinking and its biocompatibility [126]. Table 5 summarizes data on antibacterial agents used within hydrogels. Natural compounds, such as ginger fraction, show efficacy against key periodontopathogens (*S. mutans*, *P. gingivalis*) and can be used for the functionalization of titanium medical implants. Azithromycin (AZ) is indicated for combating endodontic infections caused by *A. actinomycetemcomitans* and *A. naeslundii*. Aloe vera (AV) is utilized in endodontics due to its activity against *E. faecalis,* combining disinfectant properties with biocompatibility. Silver nanoparticles (AgNPs) possess a broad-spectrum activity (*E. coli*, *S. aureus*) and are integrated into oral regenerative constructs, providing prolonged antimicrobial protection.

### 5.3. Description of Hydrogels with Antioxidant Effect

Hydrogels with antioxidant properties are being developed to neutralize reactive oxygen species (ROS) and reduce oxidative stress arising during photopolymerization or because of pathological conditions The study aimed to mitigate the negative effects of the photopolymerization process of gelatin methacrylate (GelMA) on mesenchymal stem cells. To preserve the socket after tooth extraction, a system was developed that can combat excess ROS, which delays the healing process. Table 6 systematizes data on antioxidant hydrogels used in dentistry. Dental Pulp Stem Cell-Conditioned Medium (DPSC-CM) containing peroxiredoxins (PRDX 1–6) and superoxide dismutase (SOD1) was integrated into GMP-grade GelMA hydrogels to mitigate photopolymerization-induced oxidative stress. This strategy improves cell viability and growth by reducing cellular oxidation. Gallic acid (GA) incorporated into a dual-network hydrogel based on GelMA and oxidized dextran (ODex) is applied for extraction socket site preservation. This hydrogel effectively eliminates excess reactive oxygen species (ROS) while simultaneously stimulating angiogenesis and osteogenesis, which is critical for bone tissue healing.

## 6. The Application of Hydrogel-Based Delivery Systems (GelMA Systems) in Dentistry

**Figure 4:**
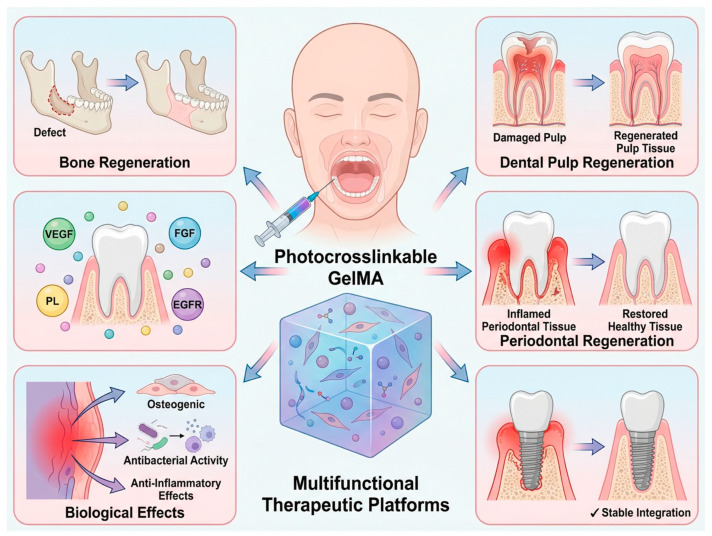
Multifunctional therapeutic effects of PC-GelMA in dentistry. The schematic illustration summarizes the therapeutic potential of PC-GelMA hydrogels as injectable biomaterials for dental tissue engineering.

PC-GelMA, owing to the excellent properties of the biopolymer, the efficiency of photopolymerization, and the ability to impart pro-angiogenic, antibacterial, anti-inflammatory properties, or combinations thereof, is applied in bone regeneration, dental pulp regeneration, and periodontal regeneration, as well as in the treatment of pulpitis, peri-implantitis, and periodontitis in dentistry, as summarized in Figure 4.

PCHs function as an integrated platform for cells and growth factors, providing support for angiogenesis, trophic restoration, and the formation of a functional pulp–dentin complex. This makes them a key tool in modern regenerative dentistry [125].

**Fundamental Principles**

A critical component of GelMA-based regenerative systems is the selection of appropriate cell populations. Human dental pulp stem cells (hDPSCs) are among the most extensively studied cell types for dental tissue engineering [126,127,129,130]. DPSCs originate from neural crest-derived tissues during embryonic development and possess the ability to differentiate into multiple lineages in vitro, including odontoclasts, osteoblasts, chondrocytes, adipocytes, myocytes, and neural cells [131]. Compared with mesenchymal stem cells obtained from other sources, such as bone marrow or adipose tissue, DPSCs exhibit a higher proliferation rate and comparable or even superior osteogenic potential. Morphologically and functionally, hDPSCs share several characteristics with mesenchymal stem cells, including fibroblast-like morphology, selective adherence to plastic surfaces, and the capacity to form colonies in vitro [132]. In the dental pulp, these stem cells reside within the connective tissue of permanent teeth and can be obtained from clinically accessible sources such as third molars or orthodontically extracted premolars [111]. DPSCs exert significant regenerative effects through paracrine signaling. They secrete a broad range of bioactive molecules, including growth factors, cytokines, and chemokines, which regulate numerous biological signaling pathways involved in tissue repair and regeneration [133]. Key immunomodulatory mediators secreted by DPSCs include IL-6, IL-8, TGF-β, hepatocyte growth factor (HGF), and indoleamine-2, 3-dioxygenase (IDO), as well as angiogenic and neurotrophic factors such as VEGF, FGF-2, PDGF, IGF-1, MCP-1, RANTES, fractalkine, BDNF, GDNF, NGF, and NT-3 [134]. These molecules collectively contribute to immunomodulation, angiogenesis, neuroprotection, and tissue regeneration. TGF-β, HGF, and IDO are capable of suppressing T-cell activation and inhibiting the proliferation of peripheral blood mononuclear cells, thereby modulating immune responses within the regenerative microenvironment [135,136].

Furthermore, the secretion of IL-6, IL-8, and TGF-β by hDPSCs has been shown to reduce Toll-like receptor-4 (TLR-4) expression during neuroinflammatory processes. In addition to soluble factors, DPSCs release extracellular vesicles (EVs), including exosomes and microvesicles, which act as carriers of proteins, lipids, and nucleic acids and mediate cell-to-cell communication within regenerative niches [134]. EVs are increasingly recognized as key mediators of the regenerative effects of DPSCs; they can regulate apoptosis, inflammation, and angiogenesis through the transfer of bioactive molecules to recipient cells.

Another important cellular component in regenerative dental constructs is the endothelial cell population. Human umbilical vein endothelial cells (HUVECs) are widely used as an endothelial cell model to promote vascularization in tissue-engineered constructs designed for dental and craniofacial regeneration [130,137]. HUVECs have been reported to express many important endothelial markers and signaling molecules associated with regulation of vascular homeostasis. Experimental studies have shown that GelMA hydrogels co-encapsulating hDPSCs and HUVECs enhance the formation of vascularized pulp-like tissue in vivo [130,138].

Other cellular components, such as odontoblast-like OD21 cells [74], vascular endothelial cells, and perivascular mesenchymal cells, may also be incorporated into hydrogel systems to better recapitulate the native cellular microenvironment. OD21 cells encapsulated in hydrogels of higher stiffness have higher spreading, proliferation, and viability [139].

In addition to cellular components, morphogenetic signaling molecules play a critical role in regulating tissue regeneration within hydrogel scaffolds. The most commonly used morphogens in dental tissue engineering are bone morphogenetic proteins (BMPs), vascular endothelial growth factor (VEGF), fibroblast growth factor-2 (FGF-2), and transforming growth factor-β (TGF-β), which can stimulate a variety of cellular activities and promote the formation of specific tissue structures even at low concentrations [140]. Hydrogels can also function as localized delivery systems for these bioactive molecules, enabling the controlled release of factors such as TGF-β1, BMP-2, VEGF, and PDGF to enhance odontogenic differentiation and support tissue regeneration [141]. BMP signaling pathways, in particular, play a crucial role in the regulation of odontoblast differentiation and dentin formation [142].

Despite the considerable regenerative potential of stem cell-based systems, several limitations remain for their clinical translation. Autologous stem cells provide high immunocompatibility but are associated with donor-dependent variability and limited scalability [143], whereas allogeneic cells may raise concerns regarding immunogenicity and regulatory approval. In addition, growth factors such as BMP-2 and VEGF exhibit limited stability and short half-lives within the inflammatory oral microenvironment [144], highlighting the importance of controlled-release hydrogel systems for maintaining therapeutic efficacy.

Platelet lysate (PL) is a multifunctional bioactive component in periodontal regeneration. It not only enhances the proliferation and activity of periodontal ligament fibroblasts but also supports the maintenance and expansion of periodontal ligament stem cells, thereby promoting tissue repair at both differentiated and progenitor cell levels when delivered via PCHs based on methacrylated hyaluronic acid. Additionally, platelet platelet-derived antimicrobial peptides such as β-lysin confer antimicrobial properties [145].

Despite the considerable promise of cell-based and bioactive factor-loaded systems, their clinical translation faces substantial hurdles. Variability in cell sources, particularly between autologous and allogeneic preparations, can lead to inconsistent therapeutic outcomes. Scalability remains a significant challenge, as the expansion of primary cells to clinically relevant numbers while maintaining their stemness and differentiation capacity requires highly standardized and costly Good Manufacturing Practice (GMP) protocols. Regulatory approval for cell-containing combination products is considerably more complex than for acellular hydrogels, demanding extensive safety and efficacy documentation. Furthermore, the high cost associated with cell isolation, expansion, and quality control may limit the widespread adoption of such advanced therapies. These considerations underscore that, while cellular and bioactive factor strategies offer unparalleled regenerative potential, their successful integration into routine dental practice will depend on overcoming these translational barriers through simplified, scalable, and cost-effective approaches.

**Figure 5:**
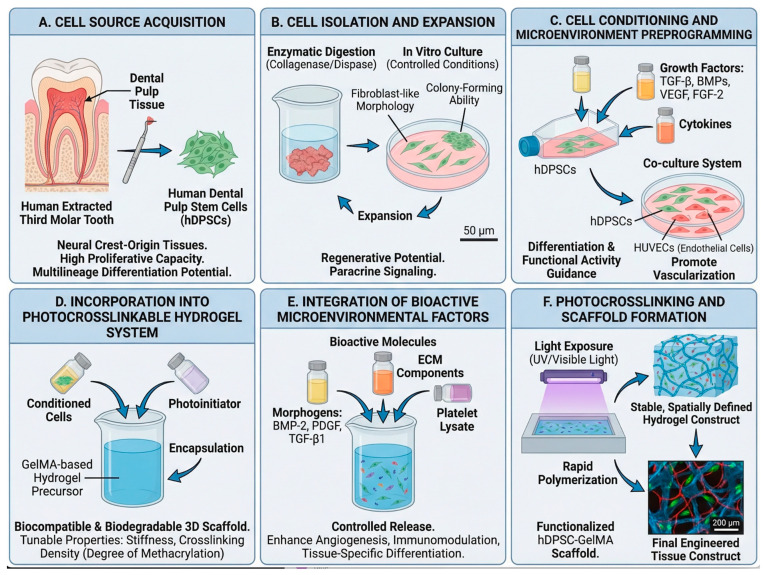
Stages of personalized delivery system formation in PCHs.

The sequential stages involved in the development of personalized cell-based delivery systems using PC-GelMA for regenerative dentistry applications: cell source acquisition, isolation and expansion in vitro, maintaining their regenerative and paracrine properties, next, during cell conditioning, growth factors and co-culture with endothelial cells guide differentiation and promote vascularization, cells are subsequently encapsulated into a GelMA-based hydrogel system along with a photoinitiator. The construct is further enhanced by the integration of bioactive factors and microenvironmental regulation; light-induced photocrosslinking leads to formation of a stable, three-dimensional scaffold containing cells and signaling molecules, resulting in a functional engineered tissue construct (Figure 5).

### 6.1. Bone Regeneration

PC-GelMA have also been explored for mineralized bone tissue regeneration through the incorporation of biomimetic mineral components that mimic the natural extracellular matrix of dentin and bone. PC-GelMA serve as delivery systems for periodontal ligament stem cells (PDLSCs), promoting the repair and regeneration of alveolar bone defects.

Pan et al. investigated GelMA hydrogels loaded with hPDLSCs for alveolar bone regeneration. In an in vivo rat critical-sized alveolar bone defect model, micro-CT analysis confirmed significant new bone formation [146].

Maxillofacial bone defects caused by fractures, tumors, and inflammation remain difficult to repair, while in vivo experiments using a 4 mm circular defect model demonstrated that 0.2 ZnSNPs/GelMA significantly enhanced new bone formation [147].

### 6.2. Dental Pulp Regeneration

Root canal therapy remains the standard treatment for necrotic permanent teeth; however, it replaces the infected pulp with biologically inert filling materials and does not restore the dentin–pulp complex [85]. Consequently, regenerative endodontic strategies aim to restore the dentin–pulp complex using biomaterial scaffolds combined with stem or progenitor cells. Hydrogels are particularly suitable for this purpose because they can be injected into the root canal space and undergo in situ crosslinking, allowing them to conform to the irregular geometry of the pulp chamber and serve as carriers for cells such as DPSCs, HUVECs, SCAP, SHED, and other mesenchymal stem cells with odontogenic potential [148,149].

From the perspective of material design, GelMA-based hydrogels are among the most widely studied systems for pulp tissue engineering. Their photocrosslinkable structure enables cell encapsulation, spatial stabilization inside the root canal, and the formation of a hydrated matrix that supports cell survival and interaction. Khayat et al. encapsulated hDPSCs and HUVECs within 5% GelMA hydrogels, followed by photocrosslinking and implantation into tooth root segments [130].

GelMA can be used in combination with other active substances for direct coverage of the tooth pulp. In its pure form, it does not have a significant mineralization potential, which makes it unsuitable for use as a standalone pulp coating material [150]. One of the most widely used ceramic additives is beta-tricalcium phosphate Ca_3_(PO_4_)_2_, which is a synthetic bone graft with osteoconductive and osteoinductive properties [151]. MTA is an alternative material for direct pulp coverage, but it has a granular structure that makes it difficult to work with. The development of an innovative fiber-based GelMA framework with the addition of TCP (tricalcium phosphate) nanoparticles can serve as a potential alternative to traditional materials for direct pulp coverage. GelMA/tricalcium phosphate scaffolds demonstrated favorable mechanical properties necessary for their intended use [151,152]. In addition, these scaffolds have sufficient biocompatibility, as evidenced by their cytocompatibility.

Several studies have demonstrated that aluminum (Al^3+^) and chromium (Cr^3+^) ions possess distinctive biological properties relevant to pulpitis therapy [153]. Specifically, they show minimal cytotoxic effects on pulp cells while also exhibiting pronounced anti-inflammatory activity. This resulted in a light-activated hydrogel (GelMA-MOF@Cr-Al) that can be used in clinical practice. The MOF@Cr-Al part can remove harmful reactive oxygen species and has strong antibacterial properties. The GelMA hydrogel quickly hardens when exposed to light, making it easy to use during dental procedures. Its three-dimensional structure also allows for a slow and controlled release of metal ions. In animal studies of pulpitis, this hydrogel significantly reduced inflammation and helped promote the formation of well-structured reparative dentin [79].

One of the most important clinical aspects of REP is the disinfection of the root canal system, as infection prevents the regeneration, restoration, and activity of stem cells [154] The effectiveness of chemical disinfection of the root canal system depends not only on the bactericidal/bacteriostatic properties of the antibacterial agents, but also on the fact that these irrigation solutions/preparations should not reduce the survival rate and proliferative capacity of the patient’s stem cell [140]. One week after the use of antibacterial drugs in the root canal, indirect negative effects were reported, such as a decrease in dentin strength and resistance to decay. These effects are mainly associated with the strong demineralizing effect and the acidic environment in which the antibiotic residues are located [155]. Silver nanoparticles (AgNPs) can be used as an endodontic disinfectant due to their broad-spectrum antibacterial properties and their lower impact on the development of microbial resistance compared with antibiotics [153,154].

Dental pulp regeneration relies on the coordinated interaction between scaffold architecture, cellular components, bioactive additives, and infection control. Current experimental evidence supports the potential of hydrogel-based systems, particularly GelMA-containing constructs, to promote vascularized pulp-like tissue formation.

### 6.3. Periodontal Regeneration

Periodontitis is a chronic plaque-associated inflammatory disease characterized by the progressive destruction of periodontal tissues and the resorption of alveolar bone [156,157]. The pathogenesis of this condition is primarily driven by pathogenic microbial biofilms that trigger a sustained host inflammatory response, ultimately leading to periodontal tissue breakdown [158,159]. Therefore, effective therapeutic strategies for periodontitis focus on reducing the bacterial burden while simultaneously promoting regenerative processes in the alveolar bone and surrounding periodontal structures.

Due to its mechanical properties, ability to support cell adhesion and proliferation, ease of administration, biocompatibility, and suitability for bioprinting, GelMA hydrogel may be a good choice for periodontal tissue regeneration [154]. GelMA hydrogel can play a role as a supporting matrix and a carrier for delivering cells and bioactive factors to the affected areas of the periodontium [154].

Several PC-GelMA-based scaffold systems demonstrate characteristics relevant for periodontal tissue regeneration, particularly through the promotion of angiogenesis, extracellular matrix formation, and controlled delivery of bioactive signals [160].

One such strategy involves GelMA scaffolds functionalized with VEGF and BMP2 mimetic peptides, which are covalently bound within a hybrid GelMA/GelNB hydrogel matrix. These peptides simultaneously stimulate angiogenesis and odontogenic differentiation, thereby creating a microenvironment favorable for regeneration of periodontal and pulp–dentin tissues [161].

For example, GelMA hydrogels containing stem cells from the apical papilla (SCAPs) and HUVECs have demonstrated the formation of interconnected microvascular networks in vitro following rapid LED-mediated photo-polymerization. In this system, SCAPs differentiate into pericyte-like cells expressing αSMA, contributing to the stabilization and maturation of newly formed blood vessels [74].

A PC-GelMA/nanohydroxyapatite (nHA) microgel system supported hPDLSC viability, proliferation, and osteogenesis, demonstrating strong potential for periodontal tissue regeneration [162].

Another emerging strategy involves the incorporation of conditioned medium derived from DPSCs into GMP-grade GelMA hydrogels, which introduces antioxidant proteins such as PRDX1–6 and SOD1 [124]. These factors help mitigate oxidative stress generated during photocrosslinking and preserve endothelial integrity, thereby improving vascularization and cell viability in vivo [124].

GelMA-Z hydrogel may represent a promising approach for the treatment of marginal periodontitis. Zeolitic imidazolate framework-8 (ZIF-8), a widely studied metal–organic framework (MOF), is composed of zinc ions (Zn^2+^) coordinated with imidazolate (Im) ligands. Owing to its ability to provide a sustained release of Zn^2+^ ions, which are known to play an important role in antibacterial activity and osteogenic regulation, ZIF-8 has attracted considerable attention for applications in various biomedical fields [159]. Through the sustained release of Zn^2+^ ions, the composite hydrogel enhanced the osteogenic differentiation of rat bone marrow mesenchymal stem cells (rBMSCs) and exhibited antibacterial activity in vitro. In a rat model, the hydrogel was further shown to decrease bacterial burden, alleviate inflammatory responses, and promote the regeneration of alveolar bone [163].

Ginsenoside Rb3 (G-Rb3), one of the principal bioactive compounds found in ginseng, has drawn significant attention from researchers due to its broad spectrum of biological and pharmacological effects [164]. Earlier studies have shown that ginsenosides possess a variety of pharmacological activities, particularly immunomodulatory and anti-inflammatory effects. In the present work, G-Rb3 was incorporated into GelMA to fabricate an injectable composite hydrogel capable of undergoing photo-crosslinking. This material was designed to regulate the inflammatory response triggered by lipopolysaccharide (LPS) in periodontal ligament stem cells (PDLSCs). Moreover, the GelMA@G-Rb3 hydrogel exhibited notable therapeutic benefits in a rat model of periodontitis [165].

By combining psoralen with GelMA, researchers fabricated a new injectable scaffold known as Pso-GelMA. The material enhanced the proliferation and osteogenic differentiation of bone marrow mesenchymal stem cells (BMSCs), partly by regulating the TGF-β1/Smad4 signaling pathway. Moreover, the scaffold exhibited inhibitory effects against the bacteria *Staphylococcus aureus* and *Fusobacterium nucleatum.* Due to its favorable injectability and biological activity, Pso-GelMA may serve as a promising material for adjunctive periodontal therapy and regeneration of damaged alveolar bone [166].

### 6.4. Peri-Implantitis

Dental implants are increasingly used as a reliable solution for replacing missing teeth. However, alongside their growing popularity, there has been a rise in inflammatory conditions affecting the tissues around implants—known as peri-implantitis. This condition is emerging as a significant public health concern, with potentially serious medical and economic implications. Consequently, there is a pressing need to develop and explore new therapeutic approaches for the management and treatment of peri-implantitis [35,167].

The authors of the article developed a sonosensitive antibacterial nanosystem simultaneously incorporating metformin (Met) and bone morphogenetic protein-2 (BMP-2), which was engineered to enhance therapeutic efficacy for peri-implantitis treatment. Zeolitic imidazolate framework-8 (ZIF-8) was utilized as a carrier for hematoporphyrin monomethyl ether (HMME) to strengthen the antibacterial effect of sonodynamic therapy. The resulting platform was further investigated in vitro with respect to its reactive oxygen species (ROS) generation capability and associated antibacterial activity [35].

These examples demonstrate the relevance of PC-GelMA systems and support the recognition of these scaffolds as promising candidates for clinical application, while also highlighting their strong potential for further research and development in regenerative medicine, particularly in bone regeneration, dental pulp regeneration, and periodontal regeneration in dentistry.

## 7. Future Perspectives and Research Directions

PC-GelMA have established themselves as a cornerstone of regenerative dentistry, owing to their unique synergy of natural polymer bioactivity and the tunable mechanical performance characteristic of synthetic materials. The capacity for rapid light-induced crosslinking using standard dental curing lamps renders these systems particularly attractive for chairside application within the confined environment of the oral cavity.

### 7.1. Integration with Advanced Bioprinting Technologies

A frontier of innovation lies in the convergence of GelMA hydrogels with portable 4D bioprinting platforms. Unlike conventional three-dimensional fabrication, 4D bioprinting introduces the dimension of time, enabling the creation of dynamic constructs that can adapt to post-implantation physiological changes. The emergence of handheld devices such as the BioPen and extrusion-based printers for GelMA bioinks facilitates the direct, intraoperative generation of patient-specific scaffolds. This approach permits precise filling of complex anatomical defects while minimizing invasiveness and shortening clinical workflows [168].

### 7.2. Multifunctional Therapeutic Platforms

Contemporary research increasingly focuses on hydrogel systems capable of executing multiple therapeutic functions simultaneously. Illustrative examples include GelMA composites incorporating metal–organic frameworks (MOFs), which exhibit triple activity—antibacterial, anti-inflammatory, and osteogenic—within a single platform [76]. Concurrently, multilayer architectures with chronologically programmed release profiles are being refined. By exploiting differential GelMA concentrations (e.g., 10% versus 20%) in adjacent layers, a gradient of crosslinking density can be engineered, yielding distinct releasetics. Such designs enable sequential stimulation of cellular events—first proliferation, then differentiation—thereby recapitulating the natural cascade of tissue repair. The integration of pH-sensitive components further sharpens this spatiotemporal control, ensuring that anti-inflammatory agents are preferentially liberated within acidic inflammatory milieus [77].

### 7.3. Overcoming Photopolymerization Constraints

Despite its advantages, the photopolymerization process itself can induce oxidative stress in encapsulated or adjacent cells, as evidenced by activation of pro-inflammatory signaling pathways. Strategies to mitigate this effect include the exploration of alternative photoinitiators that operate in the visible light spectrum (e.g., ruthenium complexes) and the employment of dityrosine crosslinking in lieu of methacryloyl-based crosslinking [169]. These approaches aim to preserve cytocompatibility without compromising crosslinking efficiency.

### 7.4. Translational Challenges and Pathways to Clinic

The clinical translation of GelMA-based technologies is encumbered by several practical hurdles. Sterilization of prefabricated hydrogels remains problematic, as conventional methods (heat, ethylene oxide, gamma irradiation) may degrade the polymer network. Long-term stability in the hostile oral environment—characterized by constant humidity, enzymatic activity, and mechanical loading—requires rigorous validation. Regulatory approval for combination products (hydrogel plus cells/drugs) adds further complexity. A promising way forward involves the establishment of standardized manufacturing protocols and the creation of well-characterized GelMA libraries, which would facilitate regulatory review. Moreover, the integration of machine learning algorithms to predict optimal bioink formulations for specific clinical indications holds potential to accelerate both development and customization.

Despite the considerable regenerative potential of stem cell- and growth factor-loaded hydrogels, several translational barriers still limit their widespread clinical implementation in dentistry. One of the major challenges is the variability of cell sources. Autologous stem cells provide high immunocompatibility but require individualized isolation and expansion, increasing treatment cost and complexity [143,170]. In contrast, allogeneic cells offer improved scalability but raise concerns regarding immunogenicity, donor variability, and regulatory approval. Another important limitation is the instability of growth factors such as BMP-2, VEGF, and TGF-β, which are characterized by short half-lives and rapid degradation in the inflammatory oral microenvironment [171,172]. Therefore, controlled and stimuli-responsive release systems are essential to improve therapeutic efficacy and reduce adverse effects associated with burst release. In addition, the manufacturing of cell-containing hydrogel systems under GMP conditions remains challenging because of the need for standardized cell expansion, sterility control, and batch-to-batch reproducibility, all of which substantially increase production costs and complicate large-scale clinical translation [173]. Regulatory and ethical issues associated with combination products containing cells and biomolecules further limit clinical implementation. Consequently, increasing attention is being directed toward cell-free regenerative strategies, including extracellular vesicles, conditioned media, platelet lysates, and acellular bioactive hydrogels, which may provide improved scalability, lower cost, and a more feasible regulatory pathway for future dental applications [174].

Addressing these challenges will unlock the full potential of PC-GelMA in dentistry, enabling a paradigm shift from palliative treatment toward true functional tissue restoration. The continued evolution of these materials—from passive scaffolds to intelligent, therapeutically autonomous systems—promises to redefine the standards of care in endodontics, periodontology, and oral surgery.

## 8. Discussion

This review demonstrates that PCHs in dentistry have undergone a substantial conceptual transition—from passive scaffolds to multifunctional, stimuli-responsive therapeutic platforms. Despite these advances in material design, a critical appraisal indicates that clinical translation remains constrained by biological uncertainty, technological complexity, and regulatory challenges.

A major strength of contemporary hydrogel systems lies in their reported biocompatibility and bioactivity [11,77,122,169,175,176,177]. However, much of this evidence is derived from simplified in vitro experiments or short-term in vivo models, which do not adequately capture the complexity of the oral environment. In particular, the long-term effects of degradation products, repeated exposure to photoinitiators, and sustained immune modulation remain insufficiently characterized. While immunomodulatory hydrogels may attenuate inflammation [159], excessive immune suppression could compromise host defense mechanisms within the microbiologically active oral cavity.

Biofilm-associated infections continue to represent a critical limitation of current therapeutic strategies. Although advanced approaches—such as spatiotemporally controlled drug delivery, enzyme-mediated biofilm disruption, and photodynamic therapy—have shown promising results [24,26,27,28,29,30], their clinical relevance is limited by the use of simplified or single-species biofilm models and the lack of validation in polymicrobial systems. Furthermore, prolonged antimicrobial exposure raises concerns regarding the development of antimicrobial resistance, particularly under subtherapeutic conditions.

Adhesion remains a key requirement for intraoral applications and has been significantly enhanced through catechol-based and mussel-inspired chemistries [178,179,180,181]. Nevertheless, most studies are conducted under static or idealized conditions, failing to account for salivary flow, enzymatic degradation, and cyclic mechanical loading. Consequently, the long-term stability and reliability of hydrogel adhesion in clinical settings remain uncertain. In addition, increasing crosslinking density to improve mechanical performance may adversely affect cellular infiltration and matrix remodeling, reflecting a persistent trade-off between structural integrity and biological functionality.

Hybrid hydrogel systems, particularly those combining GelMA with synthetic polymers such as PEGDA, offer a conceptually attractive strategy to reconcile the inherent trade-off between mechanical stability and biological functionality. By integrating the cell-adhesive and enzyme-degradable motifs of GelMA with the tunable stiffness and structural integrity of PEGDA, such composites hold promise for load-bearing applications like guided bone regeneration and periodontal defect filling [92,107,182]. Preliminary studies have demonstrated encouraging results, including enhanced mechanical properties and favorable cell responses. However, the translation of PEGDA-based hybrids into dentistry remains in its early stages. Among the publications included in this review, only a limited number address PEGDA-containing systems in the oral context, and most are restricted to preliminary in vitro characterization or short-term animal studies. This relative scarcity of evidence, combined with the increased compositional complexity of hybrids—such as the ratio of the two polymer phases, the homogeneity of the interpenetrating network, and potential phase separation during photopolymerization—necessitates further systematic investigation before definitive conclusions can be drawn regarding their long-term biocompatibility, degradation behavior, and clinical performance. Nonetheless, the conceptual appeal and early positive data position hybrid hydrogels as a promising avenue for next-generation dental biomaterials, warranting continued research efforts to establish robust synthesis protocols, elucidate structure–property relationships, and validate their safety and efficacy in clinically relevant models.

The regenerative capacity of hydrogel systems is often emphasized; however, current strategies remain overly reductionist. Although the incorporation of growth factors, exosomes, and stem cells enhances regenerative healing [39,53,130,183,184,185,186,187], these approaches frequently fail to replicate the spatiotemporal orchestration of native tissue healing. Moreover, cell-based systems introduce additional barriers, including variability in cell sources, scalability issues, regulatory complexity, and high costs, which limit their translational potential.

The versatility of PCHs as drug delivery platforms extends to a wide range of pharmacological agents. Beyond conventional small-molecule antibiotics (e.g., metronidazole, azithromycin, chlorhexidine) and anti-inflammatory drugs (e.g., ibuprofen, dexamethasone), these hydrogels can accommodate macromolecular therapeutics such as peptides, growth factors (BMP-2, VEGF, FGF-2), extracellular vesicles, and even nucleic acids (siRNA, miRNA) [188]. Encapsulation within a three-dimensional polymer network protects labile molecules from rapid enzymatic degradation in the oral cavity and enables sustained, localized delivery. However, the photopolymerization process itself poses a potential risk to sensitive biologics. Free radicals generated during crosslinking can induce oxidative damage to proteins, nucleic acids, and lipid-based vesicles, potentially compromising their bioactivity. Moreover, the exothermic nature of the curing reaction may lead to thermal denaturation of growth factors or enzymes. Strategies to mitigate these effects include the use of radical scavengers (e.g., vitamin E), the employment of more cytocompatible visible-light initiators such as LAP, and the spatial separation of the therapeutic payload from the crosslinking zone via encapsulation within nanoparticles or microspheres prior to incorporation into the hydrogel matrix [64,185]. The successful delivery of sensitive biologics therefore depends on a careful balance between achieving adequate crosslinking density and preserving the functionality of the cargo.

From a therapeutic perspective, several pharmacological classes are particularly well-suited for delivery via PCHs in dental applications. Antibacterial agents (e.g., silver nanoparticles, chlorhexidine, metronidazole, azithromycin) remain the most extensively studied, given the central role of biofilm-associated infections in endodontic and periodontal [117,189,190,191]. Anti-inflammatory drugs (e.g., ibuprofen, dexamethasone, baicalein) are essential for modulating the host response during vital pulp therapy and periodontitis, where excessive inflammation impedes regeneration [125,127,128]. Analgesics could offer post-operative pain control directly at the surgical site. Pro-angiogenic and osteogenic factors (e.g., VEGF, BMP-2, peptides, exosomes) are critical for pulp and bone regeneration, yet their delivery is complicated by instability and the need for spatiotemporal [145,185,186].

Despite this broad potential, the use of PCHs imposes specific limitations on the choice of pharmacological agents. First, the presence of reactive oxygen species (ROS) generated during photopolymerization can oxidize redox-sensitive drugs (e.g., thiol-containing compounds, catecholamines) or induce degradation of certain antibiotics [124]. Second, the drug molecule may interfere with the radical chain reaction, either by quenching radicals (thereby reducing crosslinking efficiency) or by participating in side reactions that alter its own chemical structure. Third, hydrophobic drugs may not disperse uniformly in the aqueous GelMA precursor solution, leading to phase separation and heterogeneous release. Fourth, the high water content of hydrogels favors hydrophilic compounds, whereas poorly soluble drugs require nanocarrier encapsulation [192]. These considerations underscore that the selection of an active pharmaceutical ingredient must be made in conjunction with the hydrogel formulation parameters to ensure both therapeutic efficacy and material integrity.

PCHs represent a major technological advancement [48,60,61], enabling controlled, on-demand drug release. However, each activation modality presents inherent limitations. Light-responsive systems are restricted by limited tissue penetration and potential phototoxicity; pH- and enzyme-responsive systems are affected by inter-individual variability and environmental instability [77,86]; and redox-responsive systems may exhibit unpredictable behavior due to fluctuating oxidative conditions [39,63]. While multi-responsive systems [60] offer theoretical advantages, their increased complexity may compromise reproducibility, manufacturability, and regulatory approval.

The choice of photoinitiator is a critical determinant of both the success of photopolymerization and the biological safety of the final construct. Among the most commonly used initiators, Irgacure 2959 offers high radical efficiency and moderate cytocompatibility but requires UV light (365 nm), which has limited tissue penetration and can damage DNA when used with cell-laden constructs [73,120]. LAP (lithium phenyl-2,4,6-trimethylbenzoylphosphinate) has emerged as a superior alternative for dental applications, as it is activated by visible light (405 nm), achieves high crosslinking efficiency at lower concentrations, and exhibits minimal cytotoxicity under standard curing conditions [73,121]. Camphorquinone (CQ), widely used in dental composites, is activated by visible blue light (460–480 nm) but generates ROS in the presence of co-initiators, which may contribute to oxidative stress and inflammation in adjacent pulp tissues [175].

In parallel, the selection of the external stimulus for on-demand drug release must align with the clinical context. Light-responsive systems offer high spatial and temporal precision but are constrained by light penetration depth and potential phototoxicity [67]. pH-sensitive hydrogels are attractive for periodontitis, where the inflammatory milieu is acidic, yet they may suffer from premature degradation in the fluctuating pH environment of the oral cavity [62,65]. Enzyme-responsive systems (e.g., MMP-sensitive) leverage disease-specific biomarkers but face challenges related to inter-individual variability and non-specific hydrolysis [72,193]. Redox-sensitive materials capitalize on the elevated ROS levels in inflamed or infected tissues, but their unpredictable behavior in the complex oral environment remains a concern [47,69]. Thermosensitive systems provide ease of injectability but are influenced by local temperature variations and ionic composition [37,65]. Multi-responsive platforms theoretically combine the advantages of several mechanisms; however, their increased complexity raises concerns about reproducibility, scalability, and regulatory approval [61].

Ultimately, the optimal combination of photoinitiator and stimulus-responsive mechanism must be tailored to the specific dental indication, taking into account anatomical accessibility, disease pathology, and the required duration of therapeutic action. The trend towards visible-light activatable initiators (LAP) and multi-modal stimuli responses reflects a move towards safer, more clinically adaptable platforms.

The functional classification of PCHs into anti-inflammatory, antibacterial, antioxidant, and pro-angiogenic categories, as outlined in Section 6.1, provides a useful framework for matching material properties to specific pathological features of oral diseases. However, a critical comparison reveals that the evidence base is uneven across these categories. Antibacterial hydrogels are by far the most extensively studied, reflecting the central role of biofilm-associated infections in endodontic and periodontal pathologies. Yet, the majority of these studies employ simplified mono-species biofilm models, leaving a significant gap in understanding efficacy against polymicrobial communities typical of the oral cavity. Moreover, prolonged antibacterial release raises concerns about resistance development, a risk that is seldom addressed in current literature.

Anti-inflammatory and antioxidant hydrogels, while promising for modulating host responses in pulpitis and periodontitis, often rely on a limited set of active agents (e.g., ibuprofen, dexamethasone, gallic acid). The comparative efficacy of these agents when incorporated into hydrogel matrices versus conventional delivery routes remains poorly characterized. Furthermore, the dual functionality of some systems—combining, for instance, antibacterial and anti-inflammatory actions—introduces potential antagonistic effects that have not been systematically investigated.

Pro-angiogenic hydrogels are critical for successful regeneration of vascularized tissues such as dental pulp, yet the translation of these systems is hindered by the instability of growth factors (e.g., VEGF) during photopolymerization and the difficulty of achieving spatially controlled delivery. While strategies such as peptide mimetics and exosome incorporation offer promising alternatives, their long-term safety and efficacy in large animal models remain to be established.

Thus, while the functional categorization of PCHs is conceptually useful, the depth and quality of evidence vary considerably across categories. Future research should prioritize head-to-head comparisons of different functional strategies, standardized multi-species biofilm models, and rigorous long-term safety assessments to inform clinical translation. Similarly, although prolonged and programmable drug release is widely regarded as a key advantage of hydrogel [44,188,194,195], many systems still exhibit an initial burst release followed by suboptimal sustained delivery, which may reduce therapeutic efficacy. Efforts to achieve precise temporal control often rely on increasingly complex architectures, raising concerns regarding scalability and clinical feasibility.

The physicochemical properties of hydrogels—including swelling behavior, porosity, and permeability—represent another critical challenge. While these properties are essential for nutrient diffusion and cell infiltration [96,196], excessive swelling may lead to mechanical instability, tissue compression, and uncontrolled drug release. Although composite reinforcement strategies partially mitigate these [77,145,154,169,192], they introduce additional variables that may influence degradation kinetics and biocompatibility.

Several emerging strategies directly address the limitations discussed above. Portable 4D bioprinting enables intraoperative fabrication of patient-specific scaffolds that adapt to post-implantation changes [160]. Incorporation of metal–organic frameworks (MOFs) into GelMA matrices offers sustained multi-functional release while protecting labile payloads during photopolymerization [194]. Multilayer architectures with programmed release profiles deliver temporally sequenced bioactive cues, recapitulating natural healing. Collectively, these advanced approaches hold promise for overcoming current translational barriers.

Finally, despite their considerable promise, PCHs remain largely experimental systems. Their ability to autonomously respond to pathological cues is compelling; however, issues related to signal specificity, response predictability, and long-term stability remain insufficiently resolved under clinically relevant conditions.

## 9. Conclusions

GelMA-based hydrogels are a highly versatile platform in regenerative dentistry due to their biocompatibility, tunable properties, and ability to incorporate cells and bioactive molecules. These features have enabled significant progress in periodontal, bone, and dental pulp tissue engineering. However, several limitations remain. In particular, GelMA exhibits relatively low intrinsic mechanical strength, which may restrict its use in load-bearing environments. In addition, batch-to-batch variability related to gelatin source, degree of methacrylation, and photopolymerization conditions may affect reproducibility and clinical translation. Recent studies have addressed these challenges through mechanical reinforcement strategies, including dual-network systems, polymer blending, and nanocomposite incorporation, as well as improved standardization of synthesis and crosslinking protocols to enhance batch consistency. Overall, GelMA remains a promising biomaterial for regenerative dentistry, although further optimization of its mechanical performance and manufacturing reproducibility is required to support broader clinical application.

## Figures and Tables

**Table 3:** Advantages and disadvantages of using polymer-based hydrogels.

Type of Hydrogel	Synthetic	Semi-Synthetic
Advantages	High mechanical strength, adjustable hardness, fast curing [74,81].	High adhesion, bioactivity, biocompatibility, mechanical strength, injectability and minimal invasiveness, prolonged drug delivery [74,79,85].
Disadvantages	Biological inertness, cytotoxicity of photoinitiators, limited osteoinduction [79,80].	Limited mechanical strength, Degree of Substitution instability in GelMA (Degree of Substitution—the percentage of lysine residues of gelatin substituted with methacryloyl groups) [74,85].

**Table 4:** Comparison of swelling behavior, mechanical properties, degradation, and cytocompatibility of GelMA-, PEGDA-, and hybrid-based hydrogels.

Parameter	GelMA	PEGDA	Hybrid Systems
Swelling ratio	Pristine GelMA after 24 h-87.92 ± 1.33% [104]. Decreases with increasing methacryloyl substitution and polymer concentration: ~50% (49.8%), ~30% (63.8%), ~25% (73.2%) [85]. Strong dependence on concentration: 5% GelMA- 1553 ± 30%, 10– 1223 ± 9% (96 h) [105].	Typical equilibrium swelling ratios range from ~2.5 (50 wt%) to ~3.5 (30 wt%) [106]. Swelling ratio decreases with increasing PEG molecular weight and concentration: PEG 700 (10–40% w/v): 5.88 to 2.05; PEG 3400: 9.26 to 3.60 [107].	The swelling ratio in hybrid systems is highly dependent on composition and crosslink density. Maximum swelling values (~2400–2500%) were observed in formulations with the lowest crosslink density, indicating high water uptake capacity. Increasing PEGDA content led to a pronounced decrease in swelling to ~750–800% and further to ~500–550%, corresponding to progressively higher network density. Minor composition-dependent variations were observed (~700–750%), whereas the lowest swelling values (~400–450%) were associated with the most densely crosslinked structures. Overall, these data demonstrate a strong inverse relationship between swelling ratio and crosslink density [108].
Elastic modulus	The compressive modulus of GelMA hydrogels ranges from 2.0 kPa to 30.0 kPa, depending on the degree of methacryloyl substitution and GelMA concentration. 5% GelMA without cells: 16.5 kPa. 5% GelMA without cells (12.1 kPa) [105].	77 ± 19 kPa (PEGDA) (Browning et al., 2014, J Biomed Mater Res A) [109] Mechanical properties strongly depend on crosslink density: Young’s modulus up to 3.55 ± 0.22 MPa (high PEGDA concentration) [110]. Storage modulus (G′) increases with PEGDA content and crosslink density [85].	Enhanced mechanical strength compared with pure GelMA (The stress of G10P5 was 70.6 kPa, which was almost 6 times G10 (12.1 kPa) [85].
Degradation	Pure GelMA hydrogels (10% and 20%): completely degraded after 4 weeks [85].	In vitro (NaOH): 5–10 days; In vivo: up to 12 weeks (partial), predicted full degradation ~28–30 weeks [89,111].	GelMA/PEGDA hydrogels (GelMA20% + PEGDA5% and GelMA30% + PEGDA5%): more than 50% residual weight after 4 weeks [85].
Cell viability	High cytocompatibility: >95% viability (DPSCs) [74]. Typically >80% after photopolymerization [74].	PEG-based degradable hydrogels: >90% viability within 24 h (fibroblasts) [89]. High biocompatibility: typically >87% viability [89].	Up to >138% metabolic activity after 7 days [108] >99% viability in GelMA/PEGDA systems [85].

**Table 5:** Representative antibacterial agents incorporated into hydrogels for dental applications.

Antibacterial Agent	Target Bacteria	Application
Ginger Fraction	*S. mutans*, *P. gingivalis*	Titanium medical implants [123].
Azithromycin (AZ)	*A. actinomycetemcomitans*, *A. naeslundii*	Endodontic infection control [122].
Aloe Vera (AV)	*E. faecalis*	Endodontic disinfection and healing [127].
Silver Nanoparticles (AgNPs)	*E. coli*, *S. aureus*	Oral regenerative constructs [128].

**Table 6:** Antioxidant hydrogel systems for dental applications.

Antioxidative Component	Application/Pathology	Hydrogel Type	Primary Function
DPSC-CM (PRDX) 1–6 and superoxide dismutase type 1 (SOD1) (PRDX 1–6, SOD1) [124]	Mitigation of photocrosslinking-induced stress	GMP-grade GelMA	Reduces cellular oxidation; improves cell viability and growth [124]
Gallic acid (GA)	Extraction socket site preservation	Dual-network: GelMA and Oxidized dextran (ODex)	Eliminates excess ROS; promotes angiogenesis and osteogenesis [125]

## Data Availability

No new data were created or analyzed in this study. Data sharing is not applicable to this article.

## Abbreviations

The following abbreviations are used in this manuscript:

AgNPs	Silver nanoparticles
AMP	Amorphous magnesium phosphate
AV	*Aloe vera*
BaTiO_3_	Barium titanate
BD	Biodentine
BDNF	Brain-derived neurotrophic factor
BMP	Bone morphogenetic protein
BMP-2	Bone morphogenetic protein-2
BMSCs	Bone marrow mesenchymal stem cells
CAT	Catalase
CQ	Camphorquinone
CS	Chitosan
CS-Str	Modified chitosan
DDS	Drug delivery systems
DEX	Dexamethasone
DoA	Degree of acrylation
DoM	Degree of methacryloylation
DPSCs	Dental pulp stem cells
DS	Degree of substitution
EVs	Extracellular vesicles
FGF-2	Fibroblast growth factor-2
GA	Gallic acid
GBR	Guided bone regeneration
GelMA	Gelatin methacryloyl
GMP	Good manufacturing practice
HAP	Hydroxyapatite
HDDS	Hydrogel-based drug delivery systems
hDPSCs	Human dental pulp stem cells
HGF	Hepatocyte growth factor
HNTs	Halloysite nanotubes
HUVECs	Human umbilical vein endothelial cells
IBP	Ibuprofen
IDO	Indoleamine 2,3-dioxygenase
IL	Interleukin
LAP	Lithium phenyl-2,4,6-trimethylbenzoylphosphinate
LCST	Lower critical solution temperature
LED	Light-emitting diode
LSPR	Localized surface plasmon resonance
MA	Methacrylic anhydride
MCP-1	Monocyte chemoattractant protein-1
MMP	Matrix metalloproteinase
MOF	Metal–organic framework
MTA	Mineral trioxide aggregate
NF-κB	Nuclear factor kappa B
NGF	Nerve growth factor
nHA	Nano-hydroxyapatite
NT-3	Neurotrophin-3
ODex	Oxidized dextran
PBS	Phosphate-buffered saline
PC-GelMA	Photocrosslinked gelatin methacryloyl
PCHs	Photocrosslinkable hydrogels
PDGF	Platelet-derived growth factor
PDLSCs	Periodontal ligament stem cells
PEDOT	Poly(3,4-ethylenedioxythiophene)
PEGDA	Polyethylene glycol diacrylate
PHEMA	Poly(2-hydroxyethyl methacrylate)
PL	Platelet lysate
POD	Peroxidase
PRDX	Peroxiredoxin
PVA	Poly(vinyl alcohol)
RGD	Arginine–glycine–aspartic acid
ROS	Reactive oxygen species
SCAPs	Stem cells from apical papilla
SHED	Stem cells from human exfoliated deciduous teeth
SOD	Superoxide dismutase
TEGDMA	Triethylene glycol dimethacrylate
TGF-β	Transforming growth factor beta
TLR	Toll-like receptor
TNF-α	Tumor necrosis factor alpha
UCST	Upper critical solution temperature
VEGF	Vascular endothelial growth factor
